# Efficacy and safety of respiratory syncytial virus vaccination during pregnancy to prevent lower respiratory tract illness in newborns and infants: a systematic review and meta-analysis of randomized controlled trials

**DOI:** 10.3389/fped.2023.1260740

**Published:** 2024-01-31

**Authors:** Juan Ma, Long Chen, ShiFang Tang, Yuan Shi

**Affiliations:** ^1^ Department of Neonatology, Children’s Hospital of Chongqing Medical University, Chongqing, China; ^2^ China International Science and Technology Cooperation Base of Child Development and Critical Disorders, Chongqing, China; ^3^ Chongqing Key Laboratory of Child Rare Diseases in Infection and Immunity, Chongqing, China; ^4^ Department of Neonatology, SongShan General Hospital, Chongqing, China; ^5^ Department of Neonatology, Women and Children’s Hospital of Chongqing Medical University (Chongqing Health Center for Women and Children), Chongqing, China

**Keywords:** respiratory syncytial virus, vaccination, pregnancy, newborns and infants, efficiency, meta-analysis

## Abstract

To evaluate the effectiveness and safety of respiratory syncytial virus (RSV) vaccination during pregnancy in preventing lower respiratory tract infection (LRTI) in infants and neonates, we conducted a systematic search of randomized controlled trials (RCTs) in five databases (PubMed, Embase and Cochrane Library, Web of Science, Cochrane Center Register of Controlled trial) until 1 May 2023. We performed a meta-analysis of the eligible trials using RevMan5.4.1 software. Our analysis included six articles and five RCTs. The meta-analysis revealed significant differences in the incidences of LRTI [risk ratio (RR): 0.64; 95% confidence interval (CI): 0.43, 0.96; *p* = 0.03)] and severe LRTI (RR: 0.37; 95% CI: 0.18, 0.79; *p* = 0.01) between the vaccine group and the placebo group for newborns and infants. These differences were observed at 90, 120, and 150 days after birth (*p* = 0.003, *p* = 0.05, *p* = 0.02, *p* = 0.03, *p* = 0.009, *p* = 0.05). At 180 days after birth, there was a significant difference observed in the incidence of LRTI between the two groups (RR: 0.43; 95% CI: 0.21, 0.90; *p* = 0.02). The safety results showed a significant difference in the incidence of common adverse events between the two groups (RR: 1.08; 95% CI: 1.04, 1.12; *p* < 0.0001). However, there was no significant difference observed in the incidence of serious adverse events (RR: 1.05; 95% CI: 0.97, 1.15; *p* = 0.23), common and serious adverse events (RR: 1.02; 95% CI: 0.96, 1.10; *p* = 0.23), or common and serious adverse events among pregnant women and newborns and infants (RR: 0.98; 95% CI: 0.93, 1.04; *p* = 0.52). In conclusion, maternal RSV vaccination is an effective and safe immunization strategy for preventing LRTI in postpartum infants, with greater efficacy observed within the first 150 days after birth.

## 1 Introduction

Respiratory syncytial virus (RSV) is a leading cause of hospitalization for lower respiratory tract diseases in infants worldwide. RSV is accountable for approximately 102,000 deaths annually from RSV infection worldwide, making it a prominent cause of death in infants under 6 months of age, particularly in low- and middle-income countries (1, 2). Severe lower respiratory tract illness associated with RSV is most prevalent between the months of March and June following birth. Studies have revealed that RSV is responsible for 50% of respiratory hospitalizations in children under 1 year of age, with approximately 60% of these cases affecting infants younger than 3 months (3–7). However, there is currently no approved RSV vaccine, which poses challenges in initiating timely vaccination against RSV between the ages of 3 and 6 months.

Maternal vaccination is an effective alternative to protect infants from viral infections. Vaccination of the mother results in an elevation of her antibody levels, and these antibodies are transferred to the fetus through the placenta, providing passive immunity for the first few months of the infant's life (1, 8–13). The World Health Organization has approved the vaccination of pregnant women to safeguard their babies from tetanus, diphtheria, pertussis, influenza, and SARS-CoV-2 (14–17). Based on this, we propose that maternal vaccination with RSV-associated vaccines could also be effective in protecting infants (18–21).

There have been advancements in RSV-related vaccines since the 1960s. After years of research, a breakthrough in the field of RSV vaccines has been achieved in 2022, following the successful completion of several phase III clinical trials. The membrane protein F of RSV has emerged as the primary target protein for RSV vaccine development in recent years (22–24). Previous studies have identified safety concerns with RSV vaccines in pregnant women, but the reasons for these issues are still unclear. This study aims to systematically evaluate the safety and efficacy of RSV vaccines in pregnant women, providing valuable evidence for clinical use.

## 2 Materials and methods

This systematic review and meta-analysis was registered in PROSPERO (CRD42019129316) prior to conducting the study search. It was conducted in adherence to the expected methodology of Cochrane intervention evaluation and presented in accordance with the recommendations of the preferred reporting project (PRISMA) guide for systematic review and meta-analysis (25).

### 2.1 Search strategy

A systematic retrieval was conducted on five databases (PubMed, Embase, the Cochrane Library, Web of Science, and Cochrane Center Register of Controlled trial) from their inception until 1 June 2023. The search terms used included vaccination, respiratory syncytial virus, pregnancy, newborns, and infants. More information on the search details can be found in Supplementary Tables S1–S5.

### 2.2 Selection criteria

The criteria for inclusion in the meta-analysis were as follows: (1) vaccination of pregnant women; (2) RSV vaccine administration; (3) comparison of RSV vaccine with placebo in randomized controlled trials (RCTs); and (4) reporting on the safety and efficacy of RSV. The exclusion criteria for the studies were as follows: (1) vaccination of non-pregnant women; (2) non-original studies, systematic reviews, meta-analyses, conference abstracts, letters, editorial comments, case reports, unpublished articles, and non-English articles; (3) studies involving animals or preclinical testing; (4) non-randomized controlled trials; and (5) outcomes that were not of interest.

### 2.3 Data extraction

The Cochrane Risk of Bias tool was utilized to evaluate the methodological quality of the included studies (26). The literature was independently screened by two individuals (JM and LC), and any disagreements were resolved through discussion or by involving a third person. The extracted data consisted of the first author's name, year of publication, country of study, study design, type of RCT bias risk assessment, sample size of the study subjects, grouping, baseline data, interventions, and outcome indicators. The main outcome measures encompassed medically attended lower respiratory tract illness, medically attended severe lower respiratory tract illness, adverse events in the maternal participants, severe adverse events in the maternal participants, adverse events in the infant participants, and severe adverse events in the infant participants. We conducted a thorough review of the included studies, original texts, and supplementary material to ensure that no data were overlooked.

### 2.4 Data analysis

The meta-analysis was conducted using RevMan 5.4.1 software. For categorical variables, the risk ratio (RR) was used as the effect index, while for continuity variables, the weighted mean difference (WMD) or standardized mean difference (SMD) was used. Each effect size was expressed with a 95% confidence interval (CI), and its point estimate was provided. The heterogeneity of the literature was assessed using the *χ*^2^ test. If *p* > 0.1 and *I*^2^ ≤ 50%, the fixed-effect model was employed. If *p* ≤ 0.1 and *I*^2^ > 50%, the source of heterogeneity was analyzed, and after excluding obvious clinical heterogeneity, the random-effects model was used to evaluate the source of heterogeneity. Sensitivity analysis was performed for both models. The significance level for the meta-analysis was set at *α* = 0.05, unless otherwise stated. Furthermore, a one-way sensitivity analysis was conducted to assess the impact of the included studies on the pooled outcome for outcomes with significant heterogeneity. Publication bias was assessed visually using funnel plots generated by Review Manager 5.4.1 version (Cochrane Collaboration, Oxford, UK), and Egger's regression tests were conducted using Stata 15.1 version (Stata Corp, College Station, TX, USA) for outcomes with three or more included studies. A *p*-value < 0.05 was considered statistically significant for publication bias.

## 3 Results

### 3.1 Characteristics of the included studies

**Figure 1:**
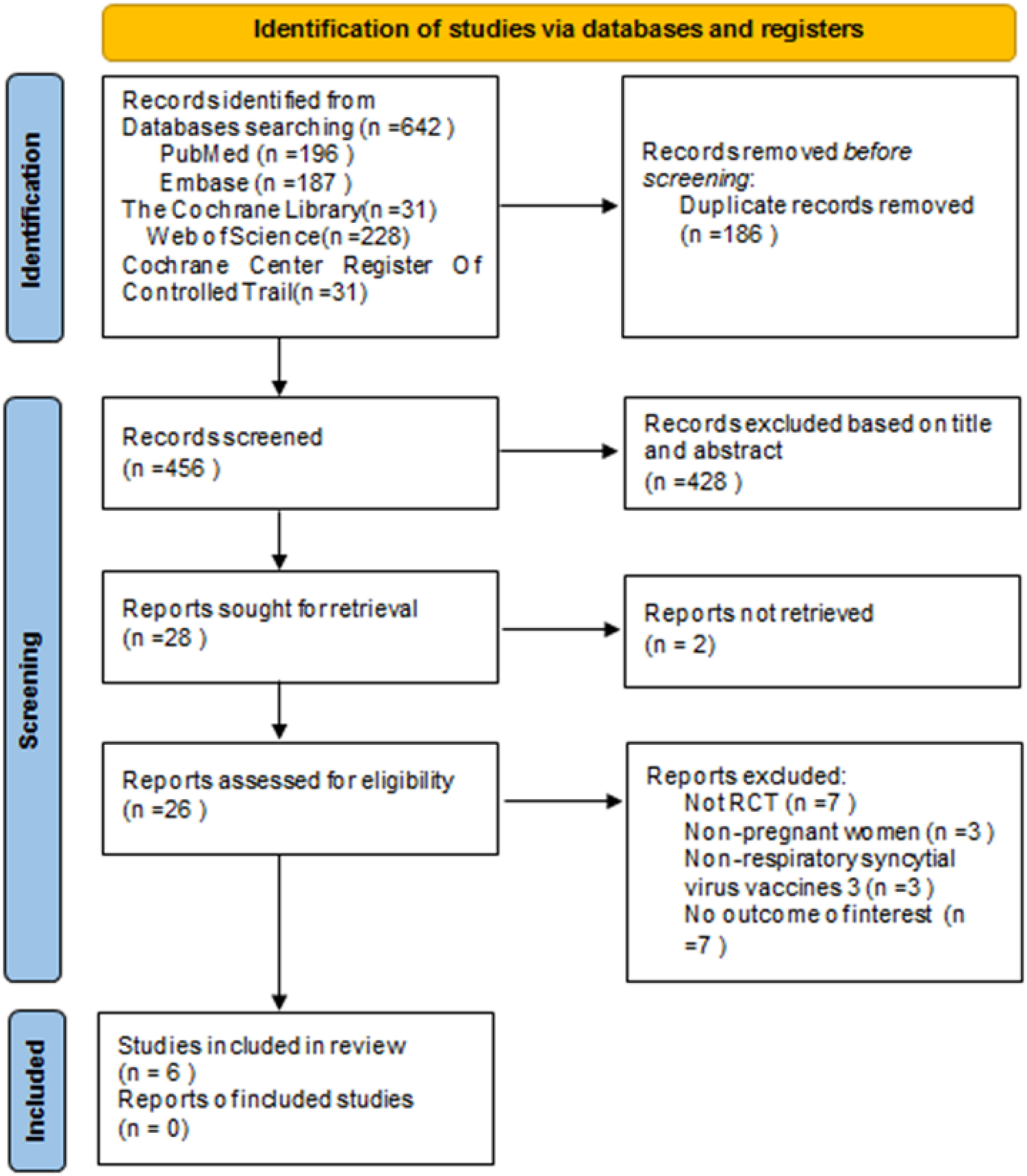
Flow chart of study identification and selection.

**Figure 2:**
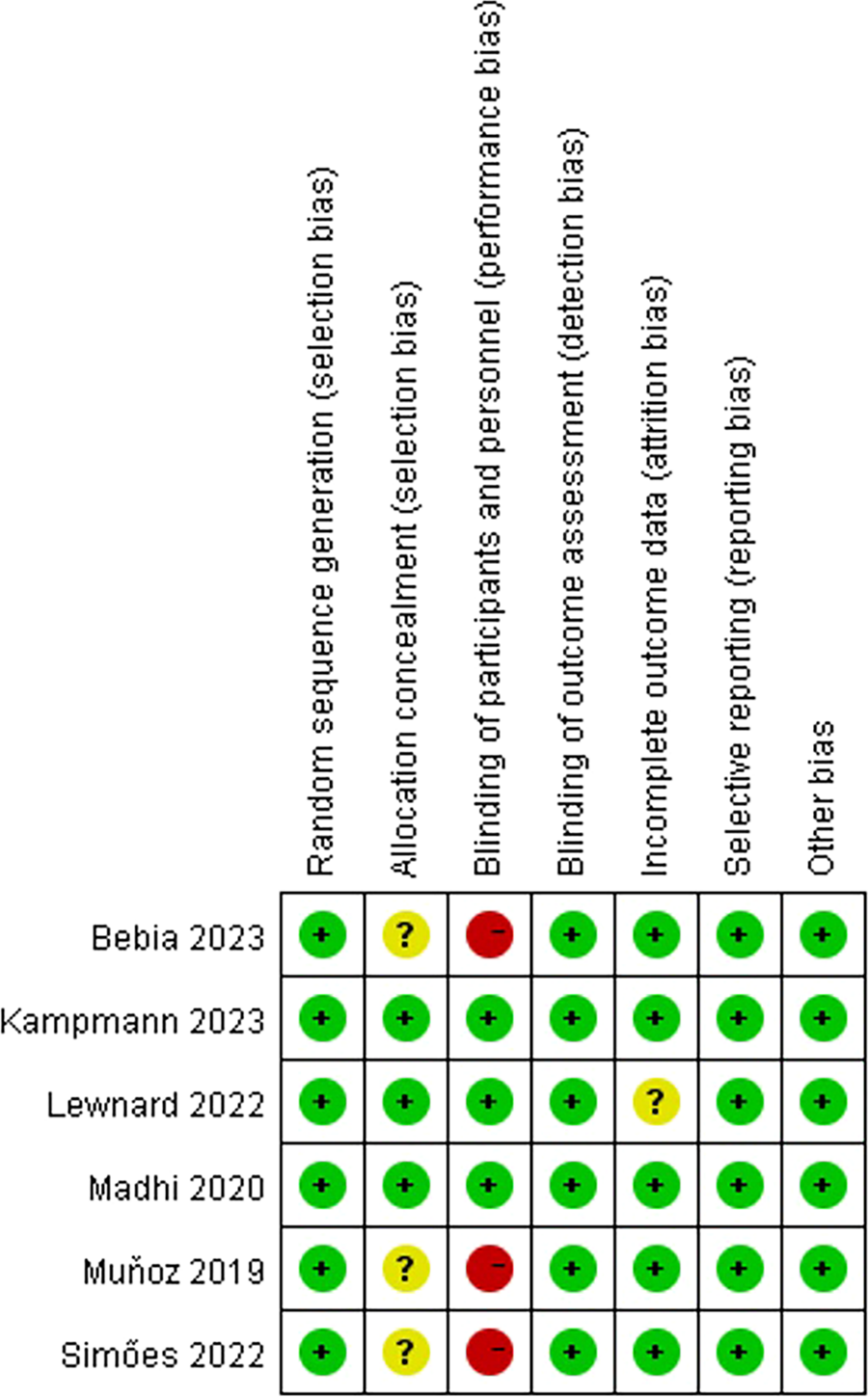
Risk of bias summary for the included RCTs (the green color and special symbol “+” represent a low risk of bias, and the yellow color and special symbol “?” represent an unclear risk of bias).

The flowchart of the system search and selection process is shown in Figure 1. A total of 642 relevant articles were obtained from PubMed (*n* = 196), Embase (*n* = 187), Cochrane Library (*n* = 31), Web of Science (*n* = 228), and Cochrane Center Register of Cochrane Controlled Trial (*n* = 31). After removing duplicates, 456 titles and abstracts were reviewed. Finally, six full-text articles were included for the pooled analysis, involving 17,230 pregnant women (10,226 vaccinated vs. 7,004 placebo) and 16,878 newborns (10,041 vaccinated vs. 6,837 placebo). Six of these studies were multicenter randomized controlled trials (27–32, 33, 34, 35). Four studies (27, 29–31) included the primary outcome index: the number of infants with lower respiratory tract infection (LRTI). Two of these studies (27, 31) also collected data on the number of infants with LRTI at 90, 120, 150, and 180 days after birth. Five articles (27–29, 31, 32) analyzed the safety indicators of vaccine use, including common and serious adverse reactions in mothers and infants. One article (30) examined the use of antibiotics after birth in infants whose mothers received RSV vaccine during pregnancy. Table 1 summarizes the characteristics of each of the included studies. We evaluated the quality of all included studies, which indicated that the studies have high quality and low risk of bias, as shown in Figure 2.

**Table 1:** Baseline characteristics of the included studies and methodological assessment.

Authors	Study period	Countries	Study design	Maternal participants	Infant participants
				Vaccine/Placebo	Vaccine/Placebo
Kampmann et al. (27)	2020–2022	18 countries	RCT	3,682/3,675	3,568/3,558
Bebia et al. (28)	2019–2021	9 countries	RCT	145/68	140/66
Simões et al. (29)	2019–2020	4 countries	RCT	327/79	325/78
Lewnard et al. (30)	2015–2018	11 countries	RCT	3005/1,573	2,978/1,546
Madhi et al. (31)	2015–2018	87 countries	RCT	3,045/1,581	3,008/1,561
Muňoz et al. (32)	2014–2015	8 countries	RCT	22/28	22/28

#### 3.1.1 Demographic characteristics of the included studies

There were no statistically significant differences observed between the two groups in terms of age at injection, BMI, gestation at injection, mode of delivery (vaginal/total), gestational age, and infant sex. However, the birth length showed a statistically significant difference (Table 2).

**Table 2:** Demographics and clinical characteristics of the included studies.

Outcomes	Studies	No. of patients	WMD or RR	95% CI	*p*-value	Heterogeneity			
		Vaccine/Placebo				Chi^2^	df	*p*-value	*I*^2^ (%)
Age at injection (years)	(1,2,3,5,6)	7,181/5,431	0.06	[−0.14, 0.25]	0.57	0.46	4	0.98	0
Gestational age at birth	(2,3,5,6)	3,539/1,755	−0.02	[−0.10, 0.07]	0.70	2.48	3	0.48	0
Gestation at injection	(1,3,5)	7,054/5,335	0.00	[−0.11, 0.11]	0.98	0.02	2	0.99	0
BMI (kg/m^2^)	(2,5,6)	3,212/1,677	0.07	[−0.23, 0.37]	0.67	0.39	2	0.82	0
Median Apgar score, 5 min	(1,2,5,6)	6,738/5,213	0.13	[−0.22, 0.47]	0.47	11,550.07	3	<0.00001	100
Length (cm)	(2,5)	3,148/1,627	−0.20	[−0.38, −0.03]	0.02	0.03	1	0.86	0
Weight (kg)	(2,5,6)	3,170/1,655	−0.01	[−0.03,0.02]	0.73	2.54	2	0.28	21
Mode of delivery in study(Vaginal)	(2,3,5,6)	2,562/1,266	1.00	[0.97, 1.04]	0.99	4.01	3	0.26	25
Infant Sex (male)	(1,2,3,4,5)	5,151/3,456	1.01	[0.98, 1.04]	0.44	3.63	4	0.46	0

BMI, body mass index; WMD, weighted mean difference; RR, relative risk; CI, confidence interval.

### 3.2 Effectiveness of the RSV vaccine

#### 3.2.1 Medically attended lower respiratory tract illness

**Figure 3:**
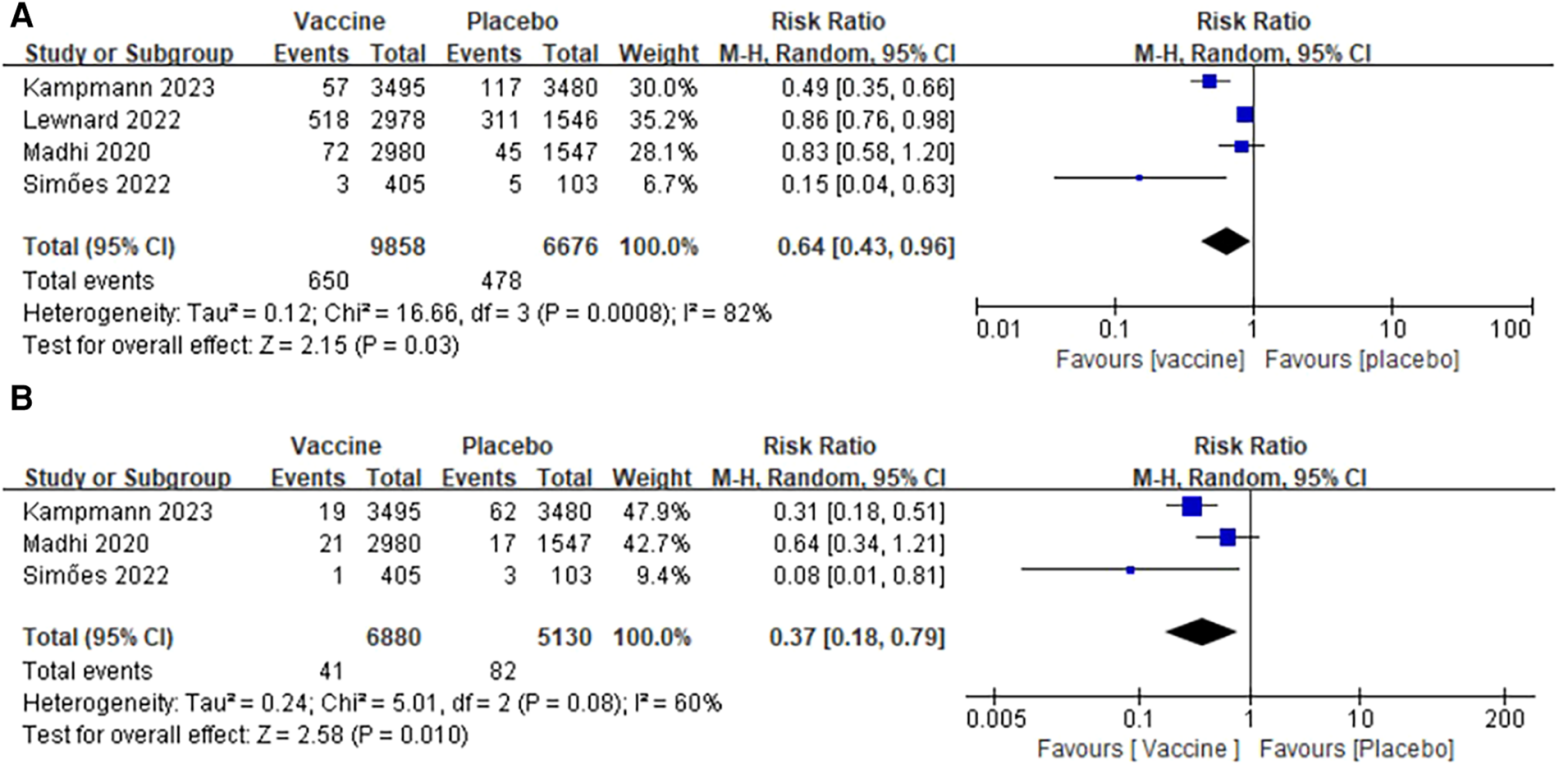
Forest plots of perioperative outcomes: (**A**) medically attended lower respiratory tract illness, (**B**) medically attended severe lower respiratory tract illness.

Data on medically attended lower respiratory tract illness were obtained from four studies involving 16,534 infants (9,858 in the vaccine group and 6,676 in the placebo group) (27, 29–31). A heterogeneity test was conducted on all the included studies, revealing a significant level of heterogeneity (*I*^2^ = 82%, *p* = 0.00008) (Figure 3A). To account for this heterogeneity, a random-effects model was used for combined analysis. The results of the meta-analysis showed that the incidence of lower respiratory tract disease in the vaccine group was significantly lower than that in the placebo group (RR: 0.64; 95% CI: 0.43, 0.96; *p* = 0.03). No publication bias was observed in the funnel plot and Egger's test (*p* = 0.217).

#### 3.2.2 Medically attended severe lower respiratory tract illness

An analysis of medically attended severe lower respiratory illness was conducted in three studies involving a total of 12,010 infants (6,880 in the vaccine group and 5,130 in the placebo group) (27, 29, 31). The pooled analysis revealed that the incidence of severe lower respiratory tract disease was significantly lower in the vaccine group compared with that in the placebo group (RR: 0.37; 95% CI: 0.18, 0.79; *p* = 0.01). However, a significant heterogeneity was observed (*I*^2^ = 60%, *p* = 0.08) (Figure 3B). After excluding the article (31), the heterogeneity test indicated a slight heterogeneity among the studies (*I*^2^ = 15%, *p* = 0.28), suggesting that the heterogeneity shift may be attributed to this specific article. No publication bias was detected in the funnel plot and Egger's test (*p* = 0.718).

#### 3.2.3 Medically attended lower respiratory tract illness (Day 90/Day 120/Day 150/Day 180)

##### 3.2.3.1 Medically attended lower respiratory tract illness (Day 90)

**Figure 4:**
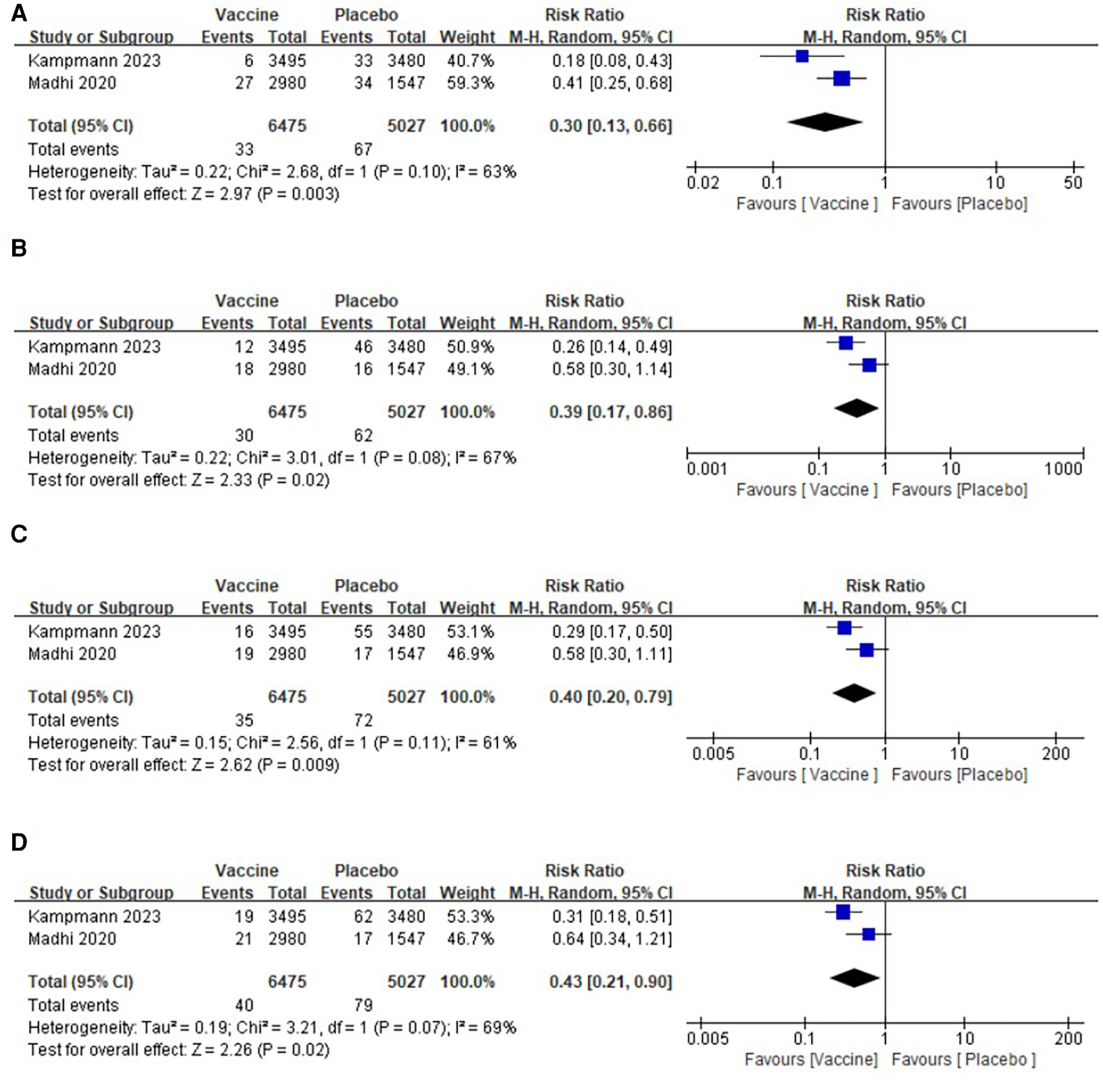
Forest plots of perioperative outcomes: (**A**) medically attended lower respiratory tract illness (Day 90), (**B**) medically attended lower respiratory tract illness (Day 120), (**C**) medically attended lower respiratory tract illness (Day 150), (**D**) medically attended lower respiratory tract illness (Day 180).

Two studies involving a total of 11,502 infants (6,475 in the vaccine group and 5,027 in the placebo group) were included in the analysis (27, 31). The pooled analysis indicated that the incidence of lower respiratory tract disease within 90 days was significantly lower in the vaccine group compared with that in the placebo group (RR: 0.30; 95% CI: 0.13, 0.66; *p* = 0.003). However, there was a significant heterogeneity observed (*I*^2^ = 63%, *p* = 0.10) as shown in Figure 4A. No publication bias was observed based on the funnel plot.

##### 3.2.3.2 Medically attended lower respiratory tract illness (Day 120)

The analysis included two articles on medically attended lower respiratory tract illness (Day 120), which involved a total of 11,502 infants. Among them, 6,475 infants were in the vaccine group and 5,027 were in the placebo group (27, 31). Heterogeneity tests were performed on all the included studies, revealing a significant heterogeneity (*I*^2^ = 67%, *p* = 0.08) (Figure 4B). A random-effects model was used for the combined analysis. The results of the meta-analysis showed that the incidence of lower respiratory tract disease in the 120-day vaccine group was significantly lower than that in the placebo group (RR: 0.39; 95% CI: 0.17, 0.86; *p* = 0.02). No publication bias was found in the funnel plot.

##### 3.2.3.3 Medically attended lower respiratory tract illness (Day 150)

Two articles were included in the analysis, reporting data on medically attended lower respiratory tract illness at Day 150 for a total of 11,502 infants. Of these, 6,475 were in the vaccine group and 5,027 were in the placebo group (27, 31). The pooled analysis revealed a significantly lower incidence of lower respiratory tract disease in the vaccine group compared with that in the placebo group (RR: 0.40; 95% CI: 0.20, 0.49; *p* = 0.009), although there was a significant heterogeneity observed (*I*^2^ = 61%, *p* = 0.11) (Figure 4C). No publication bias was detected in the funnel plot.

##### 3.2.3.4 Medically attended lower respiratory tract illness (Day 180)

Data on medically attended lower respiratory tract illness at Day 180 were obtained from two studies involving a total of 11,502 infants (6,475 in the vaccine group and 5,027 in the placebo group) (27, 31). The pooled analysis revealed a significantly lower incidence of lower respiratory tract disease in the vaccine group compared with that in the placebo group (RR: 0.43; 95% CI: 0.21, 0.90; *p* = 0.02), although there was a significant heterogeneity observed (*I*^2^ = 69%, *p* = 0.07) (Figure 4D). No publication bias was observed in the funnel plot.

#### 3.2.4 Medically attended severe lower respiratory tract illness (Day 90/Day 120/Day 150/Day 180)

##### 3.2.4.1 Medically attended severe lower respiratory tract illness (Day 90)

**Figure 5:**
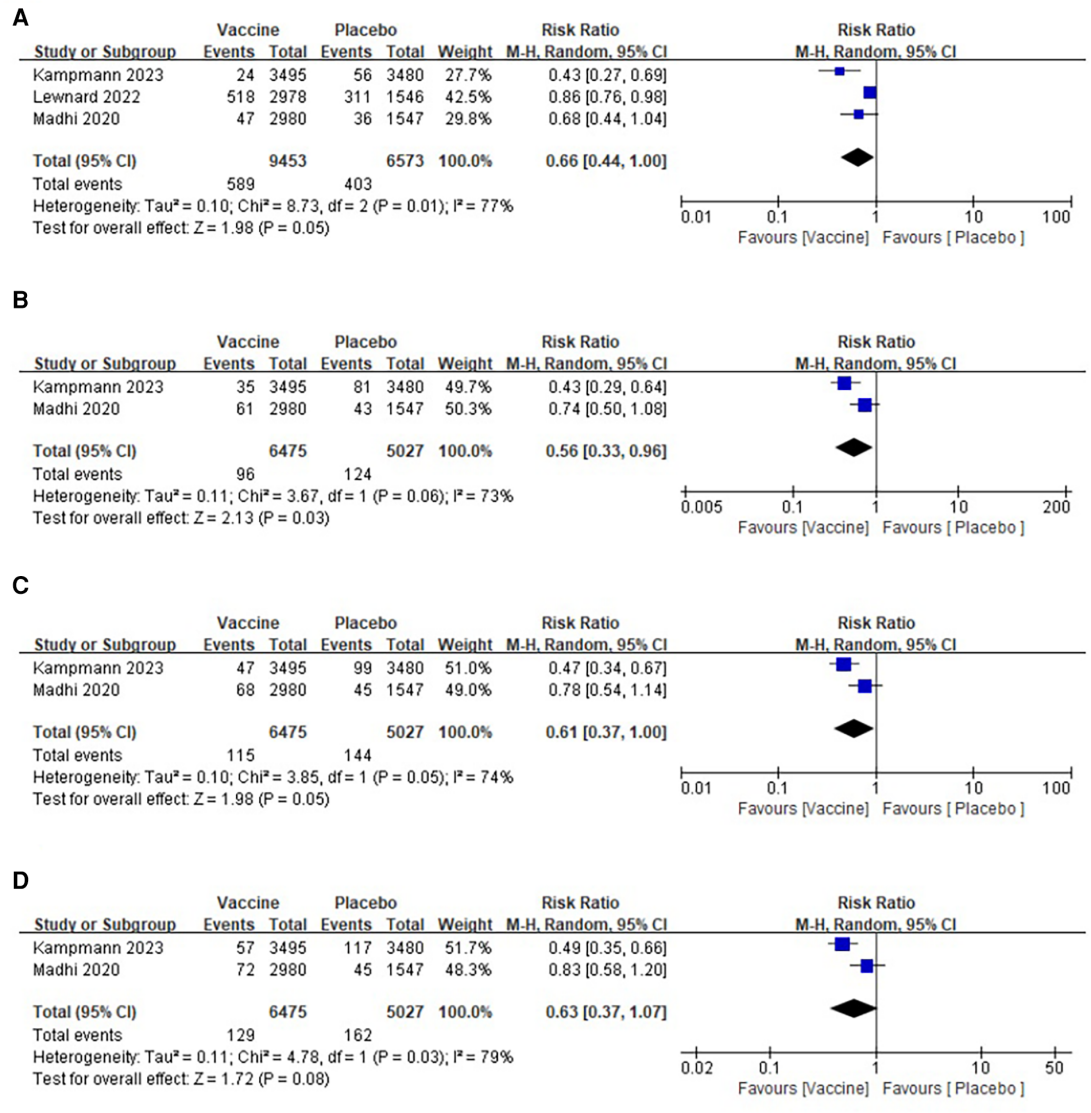
Forest plots of perioperative outcomes: (**A**) medically attended severe lower respiratory tract illness (Day 90), (**B**) medically attended severe lower respiratory tract illness (Day 120), (**C**) medically attended severe lower respiratory tract illness(Day 150), (**D**) medically attended severe lower respiratory tract illness(Day 180).

Three studies were conducted, involving a total of 16,026 infants, with 9,453 in the vaccine group and 6,573 in the placebo group (27, 30, 31). The pooled analysis revealed that the incidence of severe lower respiratory tract disease within 90 days was significantly lower in the vaccine group compared with that in the placebo group (RR: 0.66; 95% CI: 0.44, 1.00; *p* = 0.05). However, there was a significant heterogeneity observed (*I*2 = 77%, *p* = 0.01) as shown in Figure 5A. After excluding one article, the heterogeneity test indicated a slight heterogeneity among the studies (*I*^2^ = 12%, *p* = 0.19), suggesting that the presence of this article might have contributed to the observed heterogeneity. No publication bias was detected in the funnel plot, and the Egger's test showed no significant difference (*p* = 0.271).

##### 3.2.4.2 Medically attended severe lower respiratory tract illness (Day 120)

Two articles reported the data of medically attended severe lower respiratory tract illness (Day 120) in two groups, comprising a total of 11,502 infants (6,475 in the vaccine group and 5,027 in the placebo group) (27, 31). The pooled analysis revealed a significantly lower incidence of severe lower respiratory tract disease in the 120-day vaccine group compared with that in the placebo group (RR: 0.56; 95% CI: 0.33, 0.96; *p* = 0.03), although there was a significant heterogeneity observed (*I*^2^ = 73%, *p* = 0.06) as shown in Figure 5B. No publication bias was detected in the funnel plot.

##### 3.2.4.3 Medically attended severe lower respiratory tract illness (Day 150)

Two articles were included in the analysis for medically attended severe lower respiratory tract illness at Day 150, involving a total of 11,502 infants (6,475 in the vaccine group and 5,027 in the placebo group) (27, 31). A heterogeneity test was conducted on all the included studies, revealing a significant level of heterogeneity (*I*^2^ = 74%, *p* = 0.05) (Figure 5C). The random-effects model was employed for the combined analysis, and the results of the meta-analysis demonstrated a significantly lower incidence of severe lower respiratory tract disease in the 150-day vaccine group compared with that in the placebo group (RR: 0.61; 95% CI: 0.37, 1.00; *p* = 0.05). No publication bias was observed in the funnel plot.

##### 3.2.4.4 Medically attended severe lower respiratory tract illness (Day 180)

Data on medically attended severe lower respiratory tract illness at Day 180 were obtained from two studies, involving a total of 11,502 infants (6,475 in the vaccine group and 5,027 in the placebo group) (27, 31). A pooled analysis revealed no significant difference in the incidence of severe lower respiratory tract disease between the vaccine group and the placebo group at 180 days (RR: 0.63; 95% confidence interval: 0.37, 1.07; *p* = 0.08), although there was a significant heterogeneity observed (*I*^2^ = 79%, *p* = 0.03) (Figure 5D). No publication bias was detected in the funnel plot.

### 3.3 Vaccine safety (adverse events)

#### 3.3.1 Vaccine safety (adverse events and severe adverse events in the maternal participants)

##### 3.3.1.1 Vaccine safety (adverse events in the maternal participants)

**Figure 6:**
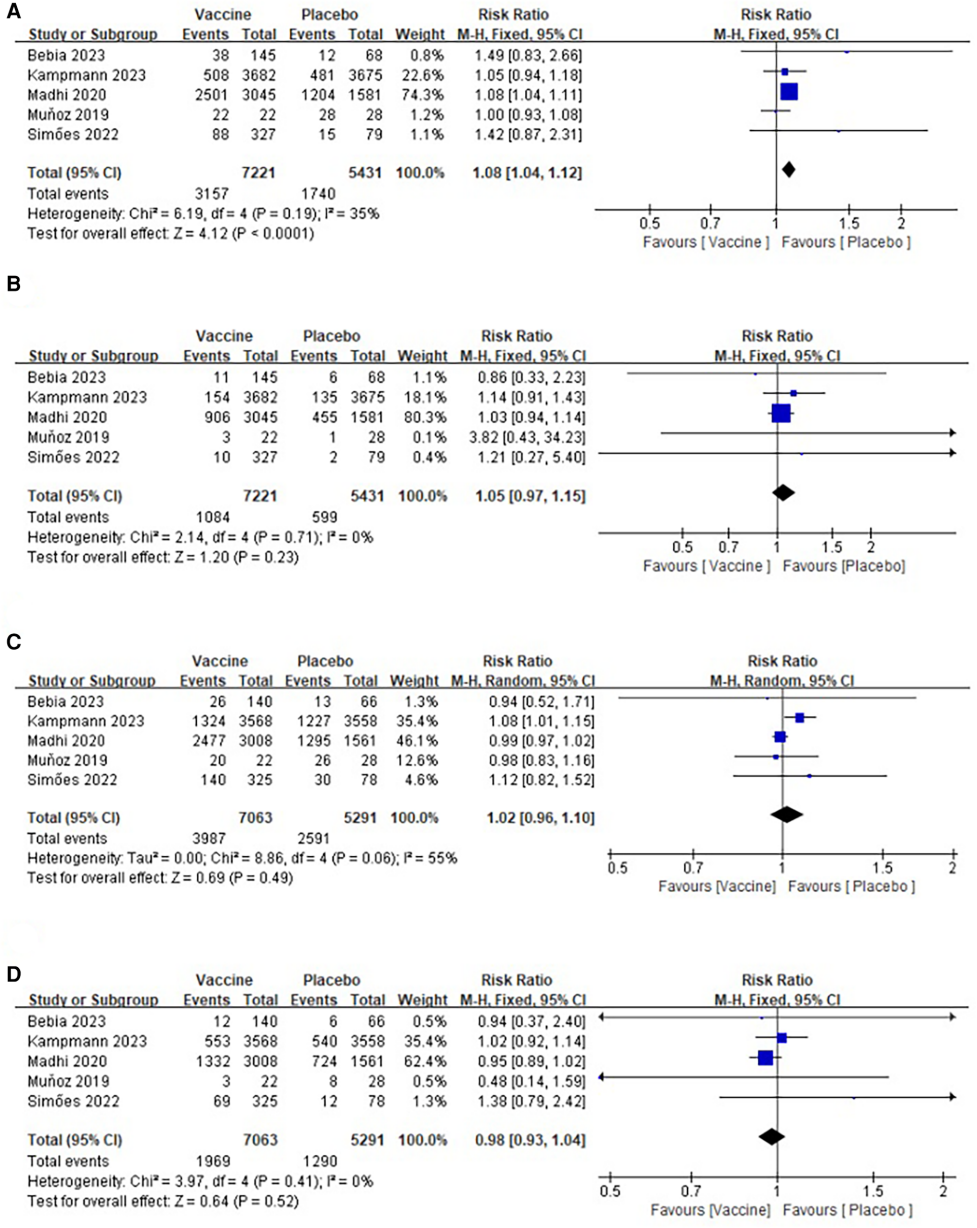
Forest plots of perioperative outcomes: (**A**) adverse events in the maternal participants, (**B**) severe adverse events in the maternal participants, (**C**) adverse events in the infant participants, (**D**) severe adverse events in the infant participants.

Five studies (27–29, 31, 32) provided data regarding common adverse events among maternal participants in the vaccine and placebo groups. The maternal rates of common adverse events were 43.72% and 32.04%, respectively. The heterogeneity test results (*I*^2^ = 35%, *p* = 0.19) (Figure 6A) indicated that a fixed-effect model was appropriate for merging the data. The merged data showed that the incidence of common adverse events was higher in vaccinated mothers (RR: 1.08; 95% CI: 1.04, 1.12; *p* < 0.0001). The visual assessment of the plots suggested a mild publication bias, but Egger's test did not show a significant difference (*p* = 0.253).

##### 3.3.1.2 Vaccine safety (severe adverse events in the maternal participants)

Data on severe adverse events in the maternal participants, including both the vaccine group and the placebo group, were obtained from five studies involving a total of 11,652 participants (7,221 in the vaccine group and 5,431 in the placebo group) (27–29, 31, 32). The pooled analysis revealed no significant difference in the incidence of severe adverse events between the two groups (RR: 1.05; 95% CI: 0.97, 1.15; *p* = 0.23), and there was no significant heterogeneity observed (*I*^2^ = 0%, *p* = 0.71) (Figure 6B). Furthermore, no publication bias was detected based on the funnel plot and Egger's test (*p* = 0.347).

#### 3.3.2 Vaccine safety (adverse events and severe adverse events in the infant participants)

##### 3.3.2.1 Vaccine safety (adverse events in the infant participants)

A total of 12,354 infants were included in five studies, with 7,063 in the vaccine group and 5,291 in the placebo group (27–29, 31, 32). The pooled analysis revealed no significant difference in the incidence of common adverse events between the two groups (RR: 1.02; 95% CI: 0.96, 1.10; *p* = 0.23). However, there was significant heterogeneity (*I*^2^ = 55%, *p* = 0.06) (Figure 6C). After excluding the article (31), the heterogeneity test showed a slight heterogeneity among the studies (*I*^2^ = 0%, *p* = 0.70), suggesting that the heterogeneity shift was related to this article. No publication bias was detected in the funnel plot and Egger's test (*p* = 0.576).

##### 3.3.2.2 Vaccine safety (severe adverse events in the infant participants)

The data from five articles were analyzed, which reported severe adverse events in the infant participants from the two groups. The study included a total of 12,354 infants, with 7,063 in the vaccine group and 5,291 in the placebo group (27–29, 31, 32). The pooled analysis indicated that there was no significant difference in the incidence of common adverse events between the two groups (RR: 0.98; 95% CI: 0.93, 1.04; *p* = 0.52), and there was no significant heterogeneity observed (*I*^2^ = 0%, *p* = 0.41) as shown in Figure 6D. The visual assessment of the plots suggested a mild publication bias, but Egger's test did not show a significant difference (*p* = 0.903).

### 3.4 Prevention of antimicrobial prescribing in infants

Only one study provided data on the use of antimicrobial prescriptions among infants (30); therefore a meta-analysis was not conducted. In a double-blind trial conducted across 11 countries, infants born to mothers who were randomly assigned to receive an experimental vaccine against RSV showed a 12.9% (95% CI: 1.3%–23.1%) decrease in the incidence of antimicrobial prescribing during the first 3 months of life compared with infants whose mothers received a placebo. The vaccine's efficacy against antimicrobial prescriptions associated with LRTI was 16.9% (95% CI: 1.4%–29.4%). These findings indicate that RSV plays a significant role in the exposure of infants to antimicrobial agents and highlight the potential of effective maternal RSV vaccines in preventing this exposure.

### 3.5 Sensitivity analysis

We conducted one-way sensitivity analyses to evaluate the influence of each individual study on the combined risk ratio (RR) for common adverse events in infant participants, LRTI, LRTIs, and LRTI (Day 90). By removing the study reported by Madhi et al. (31) in 2020, the pooled analysis of common adverse events in infant participants changed from non-significant to significant (RR: 1.06; 95% CI: 1.01, 1.13; *p* = 0.03) (Table 3A). On the other hand, when we excluded the study reported by Kampmann et al. in 2023 (1), the pooled analyses for both LRTI and LRTIs changed from significant to non-significant, respectively [RR: 0.76; 95% CI: 0.51, 1.12; *p* = 0.16 (Table 3B) and RR: 0.31; 95% CI: 0.05, 2.09; *p* = 0.23 (Table 3C)]. In addition, removing the study reported by Madhi et al. (31) in 2020 caused the pooled analysis of LRTI (Day 90) to change from significant to non-significant (RR: 0.63; 95% CI: 0.32, 1.26; *p* = 0.19) (Table 3D). In summary, the sensitivity analysis, based on the above metrics, suggests that the results are unstable.

**Table 3:** Sensitivity analysis of (A) adverse events in the infant participants, (B) LRTI, (C) LRTIs, and (D) LRTI ((Day 90).

(A)			
Study omitted	Estimate	[95% CI]	
Bebia 2023	1.0262862	.95246923	1.105824
Kampmann 2023	.99309498	.96625739	1.020678
Madhi 2020	1.0644673	1.0054245	1.1269774
Muhoz 2019	1.0329605	.95172089	1.1211349
Simöes 2022	1.0201585	.94824064	1.0975308
Combined	1.024568	.9564222	1.0975693
(B)			
Study omitted	Estimate	[95% CI]	
Kampmann 2023	.75690234	.51137823	1.1203079
Lewnard 2022	.52508193	.2914046	.946145
Madhi 2020	.54184026	.29532063	.99414283
Simöes 2022	.71188378	.49480492	1.0241985
Combined	.63983908	.42624341	.96047009
(C)			
Study omitted	Estimate	[95% CI]	
Kampmann 2023	.31476837	.04733396	2.0931931
Madhi 2020	.26214486	.11637959	.59048086
Simöes 2022	.43149492	.20800325	.89511997
Combined	.37138573	.17521691	.78718067
(D)			
Study omitted	Estimate	[95%CI]	
Kampmann 2023	.83754909	.71285236	.98405862
Lewnard 2022	.54411751	.34558713	.8566981
Madhi 2020	.63125467	.31631967	1.2597462
Combined	.66103421	.43840971	.99670746

## 4 Discussion

A total of six articles were included in this meta-analysis, with five of them being randomized controlled trials. The risk of literature bias was found to be low, and the quality score of the included studies was high. This was evident in the proper generation of random sequences, concealment of assignment, blinding, integrity of outcome data, and reporting of the study results. The meta-analysis focused on two main aspects: the effectiveness of maternal RSV vaccination in preventing lower respiratory tract infections in infants and the safety of RSV vaccination in pregnant women.

The results of this meta-analysis indicate that the incidence of lower respiratory tract disease was significantly lower in the vaccine group compared with that in the placebo group. RSV, which is the leading cause of LRTIs in infants and the leading cause of death in infants under 6 months of age, was effectively countered by the vaccine. The study revealed that the vaccine provided a heightened level of protection against LRTIs in infants. Furthermore, it significantly reduced the occurrence of severe lower respiratory tract disease in infants when compared with the placebo group. These findings suggest that maternal vaccination could be the most effective strategy for safeguarding infants at a young age.

To investigate the duration of vaccine efficacy, we conducted a meta-analysis on the incidence of LRTI in infants and the occurrence of severe lower respiratory tract infection at different time intervals (90, 120, 150, and 180 days). The findings of this meta-analysis revealed that there was no significant disparity in the occurrence of severe lower respiratory tract disease between the vaccine group and the placebo group at 180 days. However, when comparing different time intervals, the vaccine group exhibited a significantly effective protective effect compared with the placebo group, particularly within the first 150 days after birth, especially against LRTIs.

Furthermore, this meta-analysis found no noteworthy difference in serious adverse reactions between the vaccine and placebo groups. Nonetheless, there was a significant disparity in the occurrence of common side effects such as local swelling, pain, and numbness. The vaccinated mothers experienced a higher incidence of these side effects, but they were mostly transient and mild. Importantly, the occurrence of severe adverse events in mothers was similar between the vaccine and placebo groups, with no statistically significant difference. Overall, this analysis suggests that the vaccine is both safe and reliable.

Only one study included in this analysis reported data on antibiotic prescribing in infants following maternal RSV vaccination. The study suggests that RSV is a significant factor in infant antimicrobial use and provides evidence that RSV infection can be effectively prevented by maternal RSV vaccination.

Research on RSV vaccines has made rapid and groundbreaking progress in recent years due to a deep understanding of the immune mechanisms of RSV and the application of structural immunology to antigen design (33–35). The main target protein for developing RSV vaccines in recent years is the RSV membrane surface protein F (36, 37). This protein, classified as a Class I membrane protein, undergoes a significant conformational transition from Pre-F to Post-F conformations during the process of mediating viral membrane fusion, thereby completing the early infection process of the virus (19, 38–41). In the 1960s, the first generation of RSV vaccines used an all-virus inactivation strategy called FI-RSV (42–46). However, the administration of this vaccine to infants and young children did not elicit a protective response and instead resulted to an enhanced respiratory disease (ERD) following subsequent respiratory infections, leading to two infant deaths (47–49). The successful resolution of the Pre-F structure in 2013 provided a new target for developing RSV vaccine. Since then, Pre-F has become the main target protein for developing RSV vaccine, leading to a rapid increase in the number of vaccine development strategies and types (50–53). The experimental vaccines included in this meta-analysis were all designed based on Pre-F, and subunit vaccines based on Pre-F are currently considered to be among the most promising vaccine candidates.

This meta-analysis has several limitations. Firstly, the inclusion of a limited number of articles and the variation in vaccine types and doses across studies may introduce heterogeneity in the results, potentially impacting the overall findings. Future large-scale studies are needed to further investigate and validate these findings. Secondly, the results of the study may be influenced by the diversity of races and regions in the included studies. Therefore, it is necessary to conduct regional and ethnic classification studies. Lastly, there was a scarcity of literature regarding the outcome indicators of lower respiratory tract infectious diseases in infants during each time period analyzed.

## 5 Conclusions

Based on current clinical evidence, this meta-analysis suggests that the efficacy and safety of the RSV vaccine in pregnant women compared with placebo is positive. However, the existing clinical data only assess the efficacy of the vaccine in preventing RSV infection within a single RSV season, and the vaccine's ability to provide protection across several seasons remains uncertain. Further studies are needed to investigate safety issues. With advancements in molecular virology, immunology, and structural biology, the immunological mechanisms of RSV infection and the molecular properties of RSV are becoming clearer. It is anticipated that there will be significant progress in RSV vaccine research and development in the coming years. Maternal vaccination is believed to be the most effective strategy in protecting infants and newborns.

Maternal RSV vaccination has been shown to be effective in preventing lower respiratory tract infections in postpartum infants, with the greatest efficacy observed within the first 150 days of life. Therefore, the administration of RSV vaccination is considered a safe and effective immunization strategy.

## Supplementary material

The following supporting information can be downloaded at: www.mdpi.com/xxx/s1, Supplementary Figures S1–S5: search details.
