# Supplementary material for: Efficacy and safety of respiratory syncytial virus vaccination during pregnancy to prevent lower respiratory tract illness in newborns and infants: a systematic review and meta-analysis of randomized controlled trials
Source: Front Pediatr. 2024 Jan 31;11:1260740. doi: 10.3389/fped.2023.1260740 (PMC10864603; doi:10.3389/fped.2023.1260740)

Table S1. Search formula in PubMed.

("Vaccination"[MeSH Terms] OR ("vaccin"[Supplementary Concept] OR "vaccin"[All Fields] OR "Vaccination"[MeSH Terms] OR "Vaccination"[All Fields] OR "vaccinable"[All Fields] OR "vaccinal"[All Fields] OR "vaccinate"[All Fields] OR "vaccinated"[All Fields] OR "vaccinates"[All Fields] OR "vaccinating"[All Fields] OR "vaccinations"[All Fields] OR "vaccination s"[All Fields] OR "vaccinator"[All Fields] OR "vaccinators"[All Fields] OR "vaccine s"[All Fields] OR "vaccined"[All Fields] OR "vaccines"[MeSH Terms] OR "vaccines"[All Fields] OR "vaccine"[All Fields] OR "vaccins"[All Fields] OR ("Vaccination"[MeSH Terms] OR "Vaccination"[All Fields] OR ("immunization"[All Fields] AND "active"[All Fields]) OR "immunization active"[All Fields]) OR ("active immunisation"[All Fields] OR "Vaccination"[MeSH Terms] OR "Vaccination"[All Fields] OR ("active"[All Fields] AND "immunization"[All Fields]) OR "active immunization"[All Fields]) OR ("active immunisations"[All Fields] OR "Vaccination"[MeSH Terms] OR "Vaccination"[All Fields] OR ("active"[All Fields] AND "immunizations"[All Fields]) OR "active immunizations"[All Fields]) OR ("Vaccination"[MeSH Terms] OR "Vaccination"[All Fields] OR ("immunizations"[All Fields] AND "active"[All Fields]) OR "immunizations active"[All Fields]))) AND ("Respiratory Syncytial Viruses"[MeSH Terms] OR ("Respiratory Syncytial Viruses"[MeSH Terms] OR ("respiratory"[All Fields] AND "syncytial"[All Fields] AND "viruses"[All Fields]) OR "Respiratory Syncytial Viruses"[All Fields] OR ("respiratory"[All Fields] AND "syncytial"[All Fields] AND "virus"[All Fields]) OR "respiratory syncytial virus"[All Fields] OR ("Respiratory Syncytial Viruses"[MeSH Terms] OR ("respiratory"[All Fields] AND "syncytial"[All Fields] AND "viruses"[All Fields]) OR "Respiratory Syncytial Viruses"[All Fields] OR ("syncytial"[All Fields] AND "virus"[All Fields] AND "respiratory"[All Fields]) OR "syncytial virus respiratory"[All Fields]) OR ("Respiratory Syncytial Viruses"[MeSH Terms] OR ("respiratory"[All Fields] AND "syncytial"[All Fields] AND "viruses"[All Fields]) OR "Respiratory Syncytial Viruses"[All Fields] OR ("syncytial"[All Fields] AND "viruses"[All Fields] AND "respiratory"[All Fields])) OR ("Respiratory Syncytial Viruses"[MeSH Terms] OR ("respiratory"[All Fields] AND "syncytial"[All Fields] AND "viruses"[All Fields]) OR "Respiratory Syncytial Viruses"[All Fields] OR ("virus"[All Fields] AND "respiratory"[All Fields] AND "syncytial"[All Fields]) OR "virus respiratory syncytial"[All Fields]) OR ("Respiratory Syncytial Viruses"[MeSH Terms] OR ("respiratory"[All Fields] AND "syncytial"[All Fields] AND "viruses"[All Fields]) OR "Respiratory Syncytial Viruses"[All Fields] OR ("viruses"[All Fields] AND "respiratory"[All Fields] AND "syncytial"[All Fields]) OR "viruses respiratory syncytial"[All Fields]) OR ("Respiratory Syncytial Viruses"[MeSH Terms] OR ("respiratory"[All Fields] AND "syncytial"[All Fields] AND "viruses"[All Fields]) OR "Respiratory Syncytial Viruses"[All Fields] OR ("chimpanzee"[All Fields] AND "coryza"[All Fields] AND "agent"[All Fields]) OR "chimpanzee coryza agent"[All Fields]) OR ("Respiratory Syncytial Viruses"[MeSH Terms] OR ("respiratory"[All Fields] AND "syncytial"[All Fields] AND "viruses"[All Fields]) OR "Respiratory Syncytial Viruses"[All Fields] OR ("chimpanzee"[All Fields] AND "coryza"[All Fields] AND "agents"[All Fields])) OR ("Respiratory Syncytial Viruses"[MeSH Terms] OR ("respiratory"[All Fields] AND "syncytial"[All Fields] AND "viruses"[All Fields]) OR "Respiratory Syncytial Viruses"[All Fields] OR ("coryza"[All Fields] AND "agent"[All Fields] AND "chimpanzee"[All Fields])) OR ("Respiratory Syncytial Viruses"[MeSH Terms] OR ("respiratory"[All Fields] AND "syncytial"[All Fields] AND "viruses"[All Fields]) OR "Respiratory Syncytial Viruses"[All Fields] OR ("coryza"[All Fields] AND "agents"[All Fields] AND "chimpanzee"[All Fields])))) AND ("Pregnancy"[MeSH Terms] OR ("litter size"[MeSH Terms] OR ("litter"[All Fields] AND "size"[All Fields]) OR "litter size"[All Fields] OR ("prenatal care"[MeSH Terms] OR ("prenatal"[All Fields] AND "care"[All Fields]) OR "prenatal care"[All Fields]) OR ("pseudopregnancy"[MeSH Terms] OR "pseudopregnancy"[All Fields] OR "pseudopregnancies"[All Fields] OR "pseudopregnant"[All Fields]) OR ("maternal foetal relations"[All Fields] OR "maternal fetal relations"[MeSH Terms] OR ("maternal fetal"[All Fields] AND "relations"[All Fields]) OR "maternal fetal relations"[All Fields] OR ("maternal"[All Fields] AND "fetal"[All Fields] AND "relations"[All Fields]) OR "maternal fetal relations"[All Fields]) OR ("pregnant women"[MeSH Terms] OR ("pregnant"[All Fields] AND "women"[All Fields]) OR "pregnant women"[All Fields]))) AND ("infant, newborn"[MeSH Terms] OR ("infant, newborn"[MeSH Terms] OR ("infant"[All Fields] AND "newborn"[All Fields]) OR "newborn infant"[All Fields] OR ("infants"[All Fields] AND "newborn"[All Fields]) OR "infants newborn"[All Fields] OR ("infant, newborn"[MeSH Terms] OR ("infant"[All Fields] AND "newborn"[All Fields]) OR "newborn infant"[All Fields] OR ("newborn"[All Fields] AND "infant"[All Fields])) OR ("infant, newborn"[MeSH Terms] OR ("infant"[All Fields] AND "newborn"[All Fields]) OR "newborn infant"[All Fields] OR ("newborn"[All Fields] AND "infants"[All Fields]) OR "newborn infants"[All Fields]) OR ("infant, newborn"[MeSH Terms] OR ("infant"[All Fields] AND "newborn"[All Fields]) OR "newborn infant"[All Fields] OR "newborn"[All Fields] OR "newborns"[All Fields] OR "newborn s"[All Fields]) OR ("infant, newborn"[MeSH Terms] OR ("infant"[All Fields] AND "newborn"[All Fields]) OR "newborn infant"[All Fields] OR "newborn"[All Fields] OR "newborns"[All Fields] OR "newborn s"[All Fields]) OR ("infant, newborn"[MeSH Terms] OR ("infant"[All Fields] AND "newborn"[All Fields]) OR "newborn infant"[All Fields] OR "neonatal"[All Fields] OR "neonate"[All Fields] OR "neonates"[All Fields] OR "neonatality"[All Fields] OR "neonatals"[All Fields] OR "neonate s"[All Fields]) OR ("infant, newborn"[MeSH Terms] OR ("infant"[All Fields] AND "newborn"[All Fields]) OR "newborn infant"[All Fields] OR "neonatal"[All Fields] OR "neonate"[All Fields] OR "neonates"[All Fields] OR "neonatality"[All Fields] OR "neonatals"[All Fields] OR "neonate s"[All Fields])))

S2. Search formula in Embase.

((Vaccination or (Vaccinations or Immunization, Active or Active Immunization or Active Immunizations or Immunizations, Active)) and (Respiratory Syncytial Viruses or (Respiratory Syncytial Virus or Syncytial Virus, Respiratory or Syncytial Viruses, Respiratory or Virus, Respiratory Syncytial or Viruses, Respiratory Syncytial or Chimpanzee Coryza Agent or Chimpanzee Coryza Agents or Coryza Agent, Chimpanzee or Coryza Agents, Chimpanzee)) and (Pregnancy or (Litter Size or Prenatal Care or Pseudopregnancy or Maternal-Fetal Relations or Pregnant Women)) and (Infant, Newborn or (Infants, Newborn or Newborn Infant or Newborn Infants or Newborns or Newborn or Neonate or Neonates))).af.


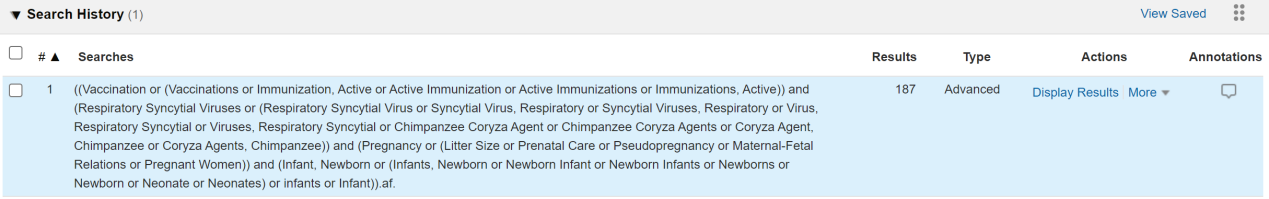


Table S3. Search formula in the Cochrane library.

((Vaccination or (Vaccinations or Immunization, Active or Active Immunization or Active Immunizations or Immunizations, Active)) and (Respiratory Syncytial Viruses or (Respiratory Syncytial Virus or Syncytial Virus, Respiratory or Syncytial Viruses, Respiratory or Virus, Respiratory Syncytial or Viruses, Respiratory Syncytial or Chimpanzee Coryza Agent or Chimpanzee Coryza Agents or Coryza Agent, Chimpanzee or Coryza Agents, Chimpanzee)) and (Pregnancy or (Litter Size or Prenatal Care or Pseudopregnancy or Maternal-Fetal Relations or Pregnant Women)) and (Infant, Newborn or (Infants, Newborn or Newborn Infant or Newborn Infants or Newborns or Newborn or Neonate or Neonates))).af.


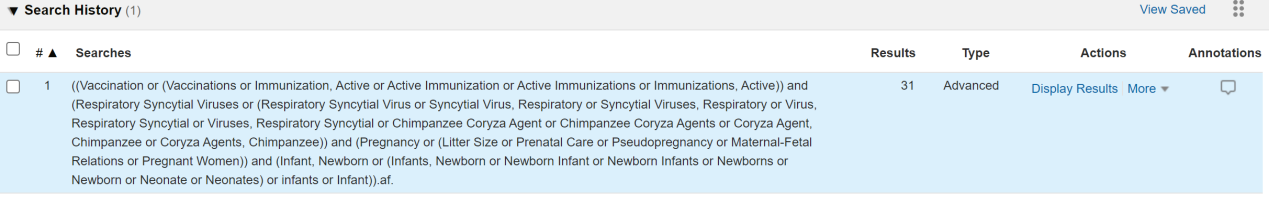


Table S4. Search formula in the Web of Science.

((((Vaccination) OR (((((Vaccinations) OR (Immunization, Active)) OR (Active Immunization)) OR (Active Immunizations)) OR (Immunizations, Active))) AND ((Respiratory Syncytial Viruses) OR (((((((((Respiratory Syncytial Virus) OR (Syncytial Virus, Respiratory)) OR (Syncytial Viruses, Respiratory)) OR (Virus, Respiratory Syncytial)) OR (Viruses, Respiratory Syncytial)) OR (Chimpanzee Coryza Agent)) OR (Chimpanzee Coryza Agents)) OR (Coryza Agent, Chimpanzee)) OR (Coryza Agents, Chimpanzee)))) AND ((Pregnancy) OR (((((Litter Size) OR (Prenatal Care)) OR (Pseudopregnancy)) OR (Maternal-Fetal Relations)) OR (Pregnant Women)))) AND ((((Infant, Newborn) OR (((((((Infants, Newborn) OR (Newborn Infant)) OR (Newborn Infants)) OR (Newborns)) OR (Newborn)) OR (Neonate)) OR (Neonates))) OR (infants)) OR (Infant)) (Topic) and Preprint Citation Index (Exclude – Database)

Table S5. Search formula in the Web of Science.

((Vaccination or (Vaccinations or Immunization, Active or Active Immunization or Active Immunizations or Immunizations, Active)) and (Respiratory Syncytial Viruses or (Respiratory Syncytial Virus or Syncytial Virus, Respiratory or Syncytial Viruses, Respiratory or Virus, Respiratory Syncytial or Viruses, Respiratory Syncytial or Chimpanzee Coryza Agent or Chimpanzee Coryza Agents or Coryza Agent, Chimpanzee or Coryza Agents, Chimpanzee)) and (Pregnancy or (Litter Size or Prenatal Care or Pseudopregnancy or Maternal-Fetal Relations or Pregnant Women)) and (Infant, Newborn or (Infants, Newborn or Newborn Infant or Newborn Infants or Newborns or Newborn or Neonate or Neonates))).af.


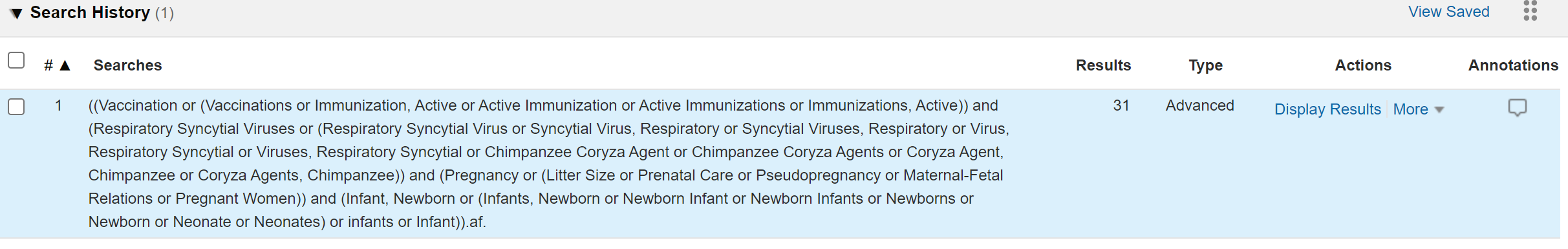

Supplement: Supplementary file 1 [file Table1.docx]
